# Daratumumab therapy in a pediatric case of C3 nephritic factor-positive proliferative glomerulonephritis with monoclonal IgG deposits

**DOI:** 10.1007/s13730-024-00868-0

**Published:** 2024-03-22

**Authors:** Sophia Giang, Anurag K. Agrawal, Aris Oates

**Affiliations:** Department of Pediatrics, University of California, San Francisco, 550 16th Street, 4th Floor, Mailstop: 3214, San Francisco, CA 94143 USA

**Keywords:** Pediatric PGNMID, Daratumumab, C3 nephritic factor

## Abstract

Proliferative glomerulonephritis with monoclonal immunoglobulin deposits (PGNMID) is an exceedingly rare cause of glomerulonephritis among children for which prognosis is generally poor, with low incidence of remission and high rates of recurrence after transplant. While there are more cases reported in the adult literature, substantial differences in pediatric vs. adult PGNMID render it essential that we further characterize pediatric cases to optimize management. We report the case of a 12-year-old male presenting initially with edema and hypertension who was subsequently diagnosed with IgG3/Kappa-dominant PGNMID. In the absence of any proven therapy and though without a detectable clone, he was empirically treated with daratumumab with positive effect to date. This is the first reported case of daratumumab monotherapy in pediatric PGNMID, as well as the first PGNMID case to detect presence of C3 nephritic factor.

## Introduction

Proliferative glomerulonephritis with monoclonal immunoglobulin deposits (PGNMID) is a recently described entity that was first reported by Nasr et al. in 2004 in a retrospectively identified series of 10 patients with proteinuria and variable renal insufficiency whose kidney biopsies shared histologic features evidencing injury from a distinct form of dysproteinemia [1]. Since then, roughly 200 cases have been reported in the literature, with the majority being in adult patients [2, 3]. Pediatric presentation is exceedingly rare, with only 15 reported cases [4–6].

PGNMID is a histologic diagnosis characterized by membranoproliferative, endocapillary proliferative, mesangioproliferative, or membranous patterns of glomerular injury caused by deposition of monoclonal IgG [1]. It is a type of monoclonal gammopathy of renal significance (MGRS) with a nonmalignant etiology for M protein-secreting B-cell or plasma cell clones [2]. MGRS are distinguished by the type of immunoglobulin (Ig) or Ig fragment that drives the pathology, which influences histologic findings. Visualization by immunofluorescence microscopy is notable for heavy and light chain-restricted deposits with IgG3 predominance. Almost all PGNMID biopsies are positive for C3 staining, with 60–80% positivity for C1q, indicating skewed activation of the alternate complement pathway. PGNMID has much lower rates of clonal isolation compared to other MGRS entities, with only 30% of cases having an identified clone as compared to ~ 70% observed in its most histologically related cousins, type I cryoglobulinemic glomerulonephritis (GN), and immunotactoid GN [3].

There are apparent differences in presentation, histology, laboratory markers, and natural progression that are emerging between adult and pediatric patients. Clinical presentation is similar in that nearly all patients have proteinuria (93–100%) and hematuria (80–90%), most of which is microscopic [2, 6]. More adults have elevated creatinine upon diagnosis compared to children (79% vs. 36%). More pediatric cases have been associated with an infectious prodrome, although this is often not characterized in adult cases [6]. Immunologically, IgG3 is the most common Ig subclass responsible for glomerular deposits in both groups, although there is a differential light chain predominance—kappa in adults and lambda in children. C3 deposition is ubiquitous in both groups (90–100%), with a majority demonstrating co-staining with C1q, although with less intensity compared to C3 immunofluorescence [2]. Hypocomplementemia is more common in pediatric compared to adult cases. Perhaps most striking is the difference in incidence of clonal isolation in adult vs. pediatric cases; while 30% of adult PGNMID cases were successful in isolating a clone, there have not been any pediatric cases to date in which the pathologic clonal population was identified [6].

PGNMID has an aggressive disease course distinguished by rapid progression toward end-stage kidney disease (ESKD) and high rates of post-transplant recurrence in both adults and children. Twenty-eight percent of adult patients reach ESKD within a mean of 3.1 years after diagnosis; for the pediatric PGNMID population, 40% develop ESKD in a mean of 5.6 years [2, 6]. Recurrence after transplant is common with incidences of 89% and 75% among adults and children, respectively [6, 7]. Said et al. characterized a cohort of 26 adult patients diagnosed with PGNMID after kidney transplant and found that the median time to recurrence was only 5.5 months [7].

## Case report

A 12-year-old male with unremarkable past medical history initially presented to his pediatrician for lower extremity edema. He was hypertensive into the 140 s/100 s on repeated blood pressure measurements. He was discharged from clinic with a plan for salt restriction, follow-up blood pressure check, and weight loss. One month later, he presented to the emergency department for progressive worsening of his edema associated with intensifying headaches and one week of dry cough.

His exam was notable for pitting edema up to the knee and ascites. Vitals revealed increased weight (+ 12 kg) and hypertension. Labs revealed urine protein to creatinine and albumin to creatinine ratios that were immeasurably high (urine protein > 600 mg/dL, albumin > 440 mg/dL, and hematuria (133 RBCs/HPF)). Creatinine at presentation was 1.17 mg/dL (eGFR 56 ml/min/1.73m^2^). There was low C3 (9 mg/dL), borderline-low C4 (15 mg/dL), and low CH50 < 12.5U/mL. He had mild hyperkalemia and hypoalbuminemia of 2.2 mg/dL. Immunological workup was negative for the following autoantibodies: ANA, centromere B, dsDNA, Smith, SS-A, SS-B, Scl70, ANCA, and GBM. He had mildly elevated DNase B of 330U/mL with normal anti-ASO titers. Infectious studies were negative, including hepatitis A, B, and C serologies and HIV screen. ESR, CRP, LDH, and haptoglobin were within normal limits.

Initial management focused on achieving euvolemia with fluid restriction, low sodium diet, and diuresis using 25% albumin and furosemide. He was given IV methylprednisolone 1 g × 3 days, followed by prednisone 60 mg with a 6-month taper. A diagnostic kidney biopsy was performed after hospital discharge. Light microscopy demonstrated glomerulonephritis with a membranoproliferative pattern. Immunofluorescence (IF) staining of the mesangium and capillary loops was IgG3/Kappa dominant. The intensity of staining on a scale of 0 to 4 + was as follows: 3 + for IgG, 1 + for IgM, and 3 + for C3. All IgG was of the IgG3 subclass, and IF staining was negative for IgA, lambda light chains, and C1q. Electron microscopy showed numerous large mesangial and subendothelial immune complex deposits as well as rare small subepithelial deposits. Podocytes were diffusely effaced, and there was prominent remodeling of the glomerular basement membrane, with frequent areas of duplication. He was subsequently started on ACE inhibition for blood pressure and proteinuric control.

Given the concern for MGRS—specifically, PGNMID—from the biopsy findings, the following labs were sent: cryoglobulin, urine and serum immunofixation studies, C3 nephritic factor (C3NeF; hemolysis-based, C3 convertase stabilization assay), complement factor H, anti-CFH, circulating immune complex (including anti-C3b), and Machaon gene panel for C3 glomerulopathy. These were positive for C3NeF only; the genetic panel was negative for CFB, CFH, CFI, MCP, C3, and CFHR5 mutations. He was initiated on immunosuppressive treatment with mycophenolate mofetil (MMF) 6 weeks after his ED presentation. Despite 6 weeks of MMF and continued ACE inhibition, he had persistent hypocomplementemia, nephrotic-range proteinuria, and elevated creatinine. Pediatric hematology/oncology was consulted for further investigation of his monoclonal gammopathy, and hematologic malignancy such as underlying lymphoma or multiple myeloma was ruled out with a normal PET scan and bilateral bone marrow aspirates and biopsies. In discussion at adult nephrology/oncology tumor board, daratumumab was recommended over rituximab due to its efficacy in clonal population targeting in adult patients with PGNMID even though there was no clone identified in this patient. Additionally, the discussion reviewed the favorable safety profile and tolerability based on experience in pediatric patients with T-cell acute lymphoblastic leukemia.

**Fig. 1:**
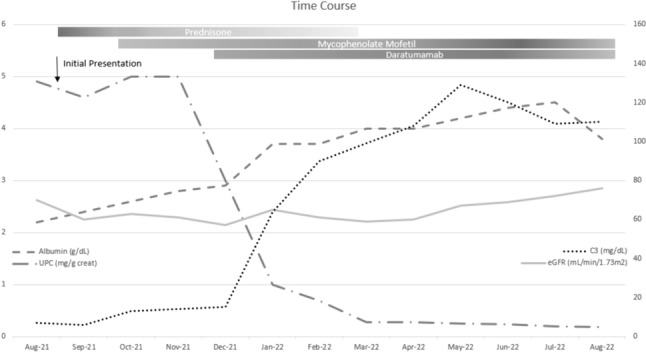
Response of various serological markers of disease activity to immunosuppressive therapies over time

Our patient began daratumumab 16 mg/kg infusions 4 months after presentation. His regimen consisted of 8 weekly infusions, followed by 8 infusions every other week and now an ongoing monthly maintenance dose. His response to daratumumab was impressive; by 1 month after the first infusion, his UPCR fell to below nephrotic-range for the first time, and his serum albumin and complement levels normalized. His C3NeF also normalized. Improvement in creatinine and cystatin C occurred more slowly, with a consistent downtrend after 6 months of therapy (creatinine 0.81 mg/dL and cystatin C 1.3 mg/L, eGFR 76 mL/min/1.73 m^2^). These continue to improve at the time of this manuscript. Changes in disease-relevant laboratory indicators as a function of time and medication changes are demonstrated in Fig. 1. The patient has had no side effects from daratumumab, though his T, B, and NK cells are quantitatively low and he has received a single dose of intravenous immunoglobulin (IVIG) for hypogammaglobulinemia. Due to his quantitative immune defect, he was empirically initiated on fungal and Pneumocystis jirovecii prophylaxis though his lymphocyte antigen and mitogen proliferation studies are currently normal indicating no qualitative immune deficit.

## Discussion

While the pathological mechanism of kidney injury via immunocomplex deposition is clear, the processes stimulating production of these rogue immunoglobulin are largely unknown. PGNMID onset is associated with infection, autoimmune disease, and solid tumor malignancies, particularly lung cancer. One hypothesis is that any trigger that activates the immune system can potentially precipitate a hypermutation event during B-cell clonal expansion that produces misfolded, nephritogenic Ig protein [2]. The IgG3 subclass, which comprises only 4–8% of circulating IgG, is by far the most frequently implicated in pediatric and adult PGNMID cases. The electrochemical and structural properties of IgG3 underlie its proclivity toward renal deposition; it is the largest of the IgG subclasses, has the most strongly positive charge and potent C1q-fixing ability, and is prone to self-aggregation through Fc-Fc interactions [3].

Treatment and outcomes in PGNMID are highly varied. Some cases spontaneously remit—as in the reports of 2 patients with parvovirus B19-associated illness [2]—while others have responded to steroids or renin-angiotensin system (RAS) inhibition alone. However, many others recur despite treatment with biologics targeting ghost B-cell or plasma cell clones. When a clone is identified, treatment is aimed at targeting that clone depending on whether it is of B-cell or plasma cell origin. Agents used for multiple myeloma and B-cell lymphoma have been repurposed in PGNMID, including bortezomib, rituximab, and daratumumab [8]. Cases that fail to identify a clone are more nuanced in their approach to treatment. RAS inhibition alone has been suggested for such cases in which patients have non-nephrotic-range proteinuria. Nephrotic patients have been treated with agents historically applied to other nephrotic syndrome etiologies including MMF, cyclophosphamide, steroids, calcineurin inhibitors, and anti-CD20 therapy (i.e., rituximab) [9]. Response rates are low and recurrence rates high [7]. Gumber et al. rationalized that the lack of isolation of a pathologic paraprotein or clone does not preclude its existence and that approach to treatment should assume presence of a clone. Ten of 16 patients did not have an identifiable clone and were empirically treated with some combination of steroids, rituximab, cyclophosphamide, and bortezomib. Seventy percent of these patients experienced complete or partial renal remission, with an additional patient responding after a second round of treatment (overall 80% response rate). Two patients experienced relapse, with one achieving no response with repeat treatment and the other still undergoing treatment at the time of publication [10].

This approach is informative for pediatric PGNMID, where none of the reported cases detected a pathologic clone. Five of the 15 pediatric patients reported in the literature were treated empirically with anti-clone agents, with 1 receiving rituximab, bortezomib, and daratumumab and 4 receiving rituximab. Xing reported 2 patients who received rituximab with stable renal function over the follow-up period (41 months for one patient; unclear timeframe for the other) [5]. The majority of 9 patients in the Miller series received steroids with or without MMF and/or a calcineurin inhibitor. Three of these patients had stable and normal renal function, although all had less than 2 years of follow-up. All patients with longer follow-up proceeded to ESKD, and 2/3 of these patients received kidney transplants with high rates of PGNMID recurrence [6].

The case here is unique in that it is the first to use daratumumab monotherapy in pediatric PGNMID. Daratumumab is an antibody against CD38 used for plasma cell depletion in relapsed or refractory multiple myeloma (MM). It mediates depletion of plasma cells, which overexpress CD38, through a wide range of mechanisms including complement- and antibody-mediated cytotoxicity, Fc*y* receptor-dependent apoptosis, and modulating immune cell composition [11]. Zand et al. conducted the first clinical trial to explore daratumumab as a treatment in PGNMID, including in treatment-naive disease. The regimen they pioneered for this open-label phase 2 trial is the one we used in our patient. As in our patient, the vast majority of enrolled subjects had nephrotic-range proteinuria, IgG/kappa-dominant deposits, and no identified clone (10/11). Of the 10 PGNMID patients who received daratumumab, 4 entered complete remission (urine protein creatinine ratio < 500 mg/day and eGFR decline < 15%), and 6 had partial remission (at least 50% reduction in baseline proteinuria and eGFR decline < 30%) by 12 months after treatment. Three patients relapsed with partial response after re-initiation of daratumumab. Most patients had obvious reduction in proteinuria by one month after their inaugural infusion [12].

Our case is also the first to document positivity for C3NeF in a patient with PGNMID. C3NeF is a group of autoantibodies against C3 convertase, a complex that is constitutively formed through binding of C3b (the hydrolyzed product of C3) to Factor B. Binding of C3Nef to C3 convertase stabilizes it against degradation and increases its convertase activity, leading to overactivation of the alternative pathway. This complement hyperactivity results in release of pro-inflammatory cytokines, recruitment of immune cells, and cytotoxicity, all of which contribute to renal injury. C3NeF levels normalized in our patient after treatment with daratumumab via elimination of B cells producing this autoantibody. C3NeF is found in the complement-mediated glomerulopathies C3 glomerulonephritis (C3GN) and dense deposits disease (DDD), as well as in a minority of healthy subjects and transiently in some patients with post-streptococcal glomerulonephritis (PSGN). C3GN has been reported in association with paraproteinemias, with rates as high as 83% [13]. Interestingly, 2 patients in the Miller pediatric case series had variants of unknown significance in genes relevant to the alternate complement pathway and presented with hypocomplementemia [6]. Complement activation via potent antibodies that may circulate at levels too low to detect using standard assays is being increasingly recognized as a driving pathophysiological mechanism in many GNs. One study found that 91% of 34 children with acute post-infectious glomerulonephritis (PIGN) had transient anti-Factor B antibodies [14]. Analogously, a significant proportion of pediatric PGNMID cases involved an infectious prodrome, which may relate to the hypocomplementemia frequently found among children but not adults with PGNMID [6].

The incomplete characterization of Ig involved in PGNMID, variability in type of Ig subclass deposited, and rare reports of conversion between C3G and PGNMID or other Ig-associated membranoproliferative glomerulonephritis all highlight the need for further studies and diagnostics to improve our precision in classifying and treating GN [15]. Doing so may help optimize therapies and improve outcomes for these GN. In this particular case, daratumumab has so far prevented progression to ESKD, with the plan to continue monthly maintenance therapy for the foreseeable future given its continued benefit and tolerability.
